# Carbolic Acid: Its Use and Abuse1Read at the quarterly meeting of the Ontario County Medical Society, at Geneva, October 8, 1901.

**Published:** 1901-12

**Authors:** George W. Sargent

**Affiliations:** Seneca Castle, N. Y.


### Carbolic Acid: Its Use and Abuse.[1]

By GEORGE W. SARGENT, M. D., Seneca Castle, N. Y.

HAVING seen something of the uses made of carbolic acid by Dr. Seneca D. Powell, and having had an accident in handling it, which would ordinarily have been somewhat serious, but under the circumstances proved to be only an interesting incident, I have attempted what I know is a very incomplete resumé of its possibilities for good and evil, especially as regards the pure acid, with some remarks on the toxicology, and the treatment in cases of poisoning. Phenol, phenyl hydrate, phenic or carbolic acid is one of our most interesting and useful remedies. There is none more capable of harm, or more commonly used accidentally, and with evil or suicidal intent. It is employed largely and carelessly by the laity, and held in somewhat superstitious regard by many, a few drops of the acid allaying all fears of infection. Every few days we hear of some accident or fatal result from it. It was formerly used as a remedy internally, and by injection. It is now employed for the purpose of a preservative in preparations of antitoxin. Considering the large doses of antitoxin now given an unsafe amount of the acid is liable to be injected with a very deleterious influence on the heart and kidneys.

1. Read at the quarterly meeting of the Ontario County Medical Society, at Geneva, October 8, 1901.

Dr. Geo. F Cott, of Buffalo, has shown that the bad results attributed to antitoxin, as nephritis and heart failure, are probably due to the carbolic acid.

In the *Medical Record* of March 11, 1899, Dr. Seneca D. Powell, of New York, asserts that alcohol is a perfect antidote to carbolic acid; moreover, that in connection with alcohol the acid is capable of greater service than generally supposed. Powell has demonstrated that the pure, or 95 per cent. acid, may be rubbed on the skin or held in the mouth with impunity until the skin or mucous membrane turns white if the parts are then rinsed with 95 per cent. alcohol. I am able to testify to the truth of this from experience, having unintentionally performed the experiment, already alluded to, on my own hands. I had on a table before me two two-ounce bottles labeled and filled respectively with carbolic acid and alcohol. Wishing to rinse my hands in the alcohol, I picked up one of the bottles, poured a liberal quantity into one palm and rubbed it over both hands as in the act of washing. I was not a little surprised to find by the greasy sensation and silvery color to which my hands were turning that I had used the carbolic acid. However, a thorough rinsing in alcohol from the other bottle, to my satisfaction, promptly restored the normal color and feeling, with the exception of a slight sensation of dryness and roughness for the rest of the day.

Since Powell's paper, this antagonism between carbolic acid and alcohol has been made considerable use of in minor surgery and in treating carbolic acid poisoning. In many instances the pathogenic germ which the physician or surgeon has to deal with is beyond control with the agents at hand in the strengths ordinarily or safely used, but carbolic acid may be employed with alcohol in full strength; and by its means a suppurative or an ulcerative process can be checked and one of healthy granulation produced quickly with rapid healing. According to the *Therapeutic Gazette* the pure acid is relatively less toxic to the normal tissues than the diluted acid, its penetrability is slight during a brief application, and its bactericidal action surpasses that of corrosive sublimate, from the fact that the action of carbolic acid is not materially affected by the presence of acids, alkalies, salts or albumin, yet in some cases may thereby be considerably increased. [Anthrax spores are destroyed by a 2 per cent. solution of carbolic acid alone in 30 days, but by a 2 per cent. carbolic with 1 per cent. hydrochloric acid, in 7 days. Staphylococcus pyogenes aureus by 1 per cent. alone in 5 minutes, but by 1 per cent. carbolic with 20 per cent. common salt in 1 minute.]

One of the most speedy and satisfactory methods of treating an indolent ulcer of the leg is carried out in the following manner: (1) curette and remove all unhealthy and false tissue; (2) apply 95 per cent. carbolic acid for a moment till the surface turns white; (3) apply 95 per cent. alcohol until the normal color of a clean granulating surface appears; (4) protect the ulcer with a covering of short, narrow strips of simple adhesive plaster, applied criss-cross so as to leave little gaps for drainage; (5) over this lay plain sterile gauze; (6) apply one or more roller bandages. This dressing applied as often as necessary will result, I have found, in a rapid cure.

Carbolic acid is serviceable in mammary tubercular and other abscesses. Here the cavity is first evacuated, then cleansed by irrigation, swabbing or curetting, and swabbed out with pure carbolic acid, then with alcohol, and finally again irrigated. In this way a normal granulating surface may be secured in a surprisingly short time. A. M. Phelps, of New York, uses the pure acid in this way in tuberculous joints. Empyema, cystitis, hydrocele, and the like, are amenable to carbolic acid, using a solution of ½ drachm to 1 drachm to the ounce of water followed with 25 or 35 per cent. alcohol.

In the case of hydrocele a quantity of the pure acid is injected and allowed to remain without further interference. Erysipelas is successfully treated by painting the affected surface with 95 per cent. acid, and when the skin turns white, with alcohol. One of our members has told me of treating a case thus, but found it very painful, though effective. Others who have reported cases make no mention of pain. A brief application of the acid to the normal skin or to an ulcer or other raw surface is not painful. Ivy poisoning is rapidly cured by a 2 per cent. lotion, and the lymphangitis that is apt to occur in connection with infected wounds is usually readily controlled by it; also the redness, pain, induration, and other signs of inflammation in caked breast or mastitis. In cellulitis of the hand or foot, Powell injects the pure acid and then alcohol, cocain being first used to avert the pain of the needle. The pure acid is used in mastoid abscesses, in septic conditions of the endometrium, ischio-rectal abscesses, furuncles, and other conditions. Some persons dread an operation and will submit to any treatment, though much slower, rather than undergo cutting. With a specially constructed syringe that will follow the sinuous tracks of fistula-in-ano it is possible to accomplish a cure without a knife.

The danger of poisoning should always be borne in mind. The long continued application of phenol might be accompanied with absorption and poisoning through the skin. I may mention the case of an infant seven days old dying from a few drops on the fingers of the nurse. The proper use of the acid does not contemplate usually protracted or continuous applications. Ignorant persons sometimes wrap the fingers or toes in the acid in full strength, or in very strong mixtures, with subsequent gangrene. There seems to be an opinion that gangrene is apt to occur when the acid is used on the extremities even in weak solution, but I think this must be a very rare result. An instance reported where two brothers, both young men, never ill previously, had gangrene of the fingers following the use of a solution containing only 1.7 per cent. carbolic acid was regarded as due to idiosyncrasy. There is no danger so far as I know from the pure acid followed with alcohol.

The treatment of poisoning cases has been very unsatisfactory. Woodman and Tidy cite twenty-one instances during a period of five years with only one recovery. The doses taken were usually one to two ounces. The smallest fatal dose I have found recorded is ½ oz. The effect of a poisonous dose is sometimes extremely rapid, but generally several hours elapse before death. The antidotes printed on the manufacturers' labels and in the books have not proven efficient. The alkalies in excess have some power, oils retard the absorption of the acid and heart stimulants, as atropine, strychnine and caffeine are useful.

In one instance 2½ grs. of caffeine dissolved in water with the aid of sodium salicylate given hypodermatically and repeated in an hour restored a man from a state of coma. Vinegar is claimed to be efficacious. Bleeding, one pound of blood being drawn, was once successful. Emetics are of no service because the stomach is benumbed or paralysed by the acid. During the past two years, since the antagonism between phenol and alcohol has been made prominent, several cases of recovery where the patients were apparently moribund have been reported after the exhibition of alcohol. In one case the stomach was washed out with pure alcohol. A 25 per cent. solution will counteract the acid on the skin and it is probable that whiskey or any strong alcoholic would answer for internal use. How this antidotal property of alcohol is exerted is not certainly known. There may be a true antagonism between the two substances, or the alcohol may act simply as a solvent, though its action seems to go farther than that. However this may be, we have the fact that alcohol will prevent and remove the caustic and poisonous effects of the acid. The primary step in poisoning cases is to wash the stomach out with alcohol, then whiskey may be given hypodermatically, with the idea of neutralising the acid in the blood. Other heart stimulants may be required. As the acid is quite rapidly taken up into the blood it has been suggested that no method of treatment is complete without bleeding. But this has not been done to any great extent, or been necessary, to my knowledge. I find four cases reported during the last eighteen months, three in the *Medical Record* and one in the *Therapeutic Gazette* of September 15, 1901. There have been others, but I am unable to refer to them at this time. In three of these, alcohol was given the undoubted credit for the recovery. In two it was the only agent used until the acuteness of the attack was over. In one, sulphate of magnesia was employed, but not considered as having had much influence on the case. In the fourth the act of drawing forward the tongue seemed to be beneficial by admitting air more freely to the lungs.

In conclusion, it seems safe to say that alcohol is the most perfect, the most certain, and the most handy antidote to carbolic acid which we possess.

